# Walking the care pathway: A phenomenological inquiry on mothers' experiences meeting the bio psychosocial needs of leukemic children

**DOI:** 10.6026/973206300220513

**Published:** 2026-01-31

**Authors:** Kannan Kasinathan, Manoharan Saravanan, Shankar Shanmugam Rajendran, Hemachitra J, Marudan Anbalagan, Nirmala Asaithambi

**Affiliations:** 1 Department of Research Scholar, Meenakshi Academy of Higher Education and Research (MAHER) (DU), Chennai & Lecturer in Nursing, College of Nursing, Madras Medical College, Chennai, Tamil Nadu, India; 2 Department of Research, Meenakshi Medical College and Research Institute & Meenakshi Academy of Higher Education and Research (MAHER - DU), Chennai - 600078, Tamil Nadu, India; 3 Department of Pediatric Nursing, College of Nursing, Madras Medical College, The TN Dr MGR Medical University, Chennai, Tamil Nadu, India; 4 Department of Hematology, Institute of Child Health and Hospital for Children & Madras Medical College, Chennai, Tamil Nadu, India; 5 Department of Child Health Nursing, College of Nursing, Madras Medical College, The TN Dr MGR Medical University, Chennai, Tamil Nadu, India; 6 Department of Obstetrics and Gynaecological Nursing, Sooriya School of Nursing, Saligramam, Chennai, Tamil Nadu, India

**Keywords:** Biopsychosocial needs, caregiving practices, childhood leukaemia, coping strategies, lived experiences, Mothers'caregiving experience, nurse-parent relationship, psycho social consequences

## Abstract

The emotional, psychosocial and practical challenges faced by mothers caring for children diagnosed with leukaemia. Therefore, it is of interest to explore the lived experiences of six mothers whose children were undergoing treatment in a tertiary-care hospital. In depth interviews were conducted and data were analyzed using a systematic qualitative framework. The study identified eight major themes, including emotional turmoil, caregiving patterns and the dynamics of healthcare relationships. Thus, we show the need for comprehensive support systems to alleviate emotional burdens and enhance caregiving capacity in pediatric oncology.

## Background:

Leukaemia is the most common childhood malignancy, accounting for a substantial proportion of paediatric cancer diagnosed globally. Acute lymphoblastic leukaemia (ALL) constitutes about nearly 75% of cases, with survival outcomes improving in a resource-rich healthcare system and which in turn leave the caregiving demand placed on families, particularly the mothers [1, 2-3], The child's treatment trajectory has been characterised by their intensive chemotherapy, prolonged hospitalisation, repeated procedures and risk of infection that creates a complex landscape for their biopsychosocial needs [4, 5]. For mothers, those caring for a child with leukaemia are considered an exhaustive journey marked by an emotional turmoil, lifestyle modification, financial pressure and the uncertainty [6]. Qualitative research has shed light on how the women deal with fear, sadness, fatigue and shifting their hope while trying to preserve a feeling of normalcy for their children. Their responsibility frequently extends beyond the physical care to those which include their emotional stabilisation, symptom monitoring, infection control practices and compliance with the demanding treatment regimens [7, 8]. In many low- and middle-income countries, families experience additional barriers such as long travel distances, limited financial resources and inadequate psychosocial support. Understanding this in hand mothers' lived experiences therefore becomes essential for designing the best targeted, culturally appropriate, nurse-led interventions [9]. Despite the growing literature on paediatric oncology caregiving, limited evidence exists on how mothers in India experience and interpret the bio psychosocial needs of their children with leukaemia. Therefore, it is of interest to explore these experiences in depth, capturing the emotional nuances, the care practices, coping strategies followed and health-system encounters that shape their caregiving journey.

## Methodology:

**Figure 1:**
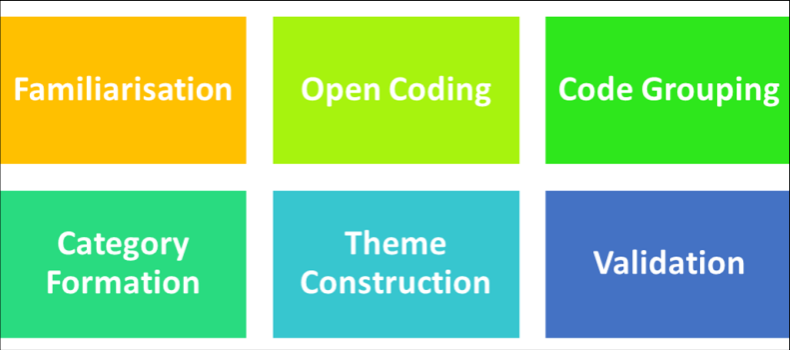
Steps of data analysis

A qualitative descriptive phenomenological approach was employed to gain an in-depth understanding of mothers' lived experiences. Six mothers of children diagnosed with leukaemia and undergoing treatment in a tertiary-care paediatric oncology unit were purposively sampled. Inclusion criteria included participants who were primary caregivers, able to communicate in Tamil or English and willing to share their experiences. Unstructured, open-ended interviews encouraged the mothers to narrate their caregiving journey. Interviews lasted for 30-45 minutes, which were audio-recorded with consent and conducted in a private hospital counselling room to ensure their comfort and confidentiality. The data Analysis followed a systematic, multistep process Figure 1. Ethical approval was obtained from the institutional review board (IEC-MMC/Approval/62042024). Informed consent, confidentiality and voluntary participation principles were maintained.

## Results:

Eight themes captured the essence of mothers' biopsychosocial experiences Table 1.

The Mothers described a whirlwind of emotions beginning with shock, disbelief and overwhelming sadness. Many of them recounted the moment of diagnosis as an emotional rupture, one of them verbalised that as it has temporarily destabilised their sense of control.

Participant Quotes: "We were crying every day."

With the time passes, hope emerged among them as the mothers observed improvements and gained clarity from their healthcare providers. Yet, a sense of fear of relapse and complications persisted till now among them.

Participant Quotes: "We believe our child will recover soon... but worry is always there."

The path to the diagnosis was chaotic, often involving the recurrent fevers, multiple blood tests, contradictory explanations and frequent emergency referrals. For many of them, the illness initially resembled common infections which they are not aware. Confirmation following the bone marrow tests elicited both the shock and relief, as mothers gained clarity.

Participant Quotes: "We thought it was dengue or typhoid."

Daily caregiving practices revolved around the infection control, nutritional vigilance and sustaining emotional warmth. Hygiene practices which included hand washing, sanitizing and restricting visitors became indispensable. Home-cooked meals were prioritised and play became a therapeutic activity preserving their children emotional normalcy.

Participant Quotes: "I use sanitizer before touching him."

Leukaemia altered the family ecosystem. Many of the mothers quit their jobs to take care of their children, struggled financially and navigated through their disrupted routines. Family roles shifted, with the grandparents, spouses, or older siblings taking on the new responsibilities. Mothers frequently felt stretched between hospital and the home tasks.

Participant Quotes: "I stopped my tailoring work."

To protect their child, the families withdrew from the family and social gatherings. Diagnosis disclosure was selective, reflecting concerns about stigma or unnecessary pity which in turn will increase their stress and depression. Some of the mothers experienced empathy and received tangible help from their relatives, offering an emotional relief.

Participant Quotes: "We keep it within the family."

Mothers cultivated certain coping mechanisms such as increasing the emotional bonding with the child, practicing mindfulness breathing and grounding routines such as cooking to maintain their psychological balance. The Hospital counselling sessions also strengthened their self-care practices.

Participant Quotes: "Cooking makes me feel better."

Nurses and doctors significantly shaped the mothers' emotional security and understanding of the treatment. Stepwise explanations from nurses reduced their anxiety, while doctors' updates facilitated their acceptance. Hospital logistics, including the ambulance transfers and ICU admissions, became the defining moments.

Participant Quotes: "The nurse cleared my doubts patiently."

Mothers closely monitored the visible improvements in their child health status that included reduced fever, better appetite, or the improved blood counts. Strict adherence to the instructions was perceived essential for the positive outcomes. However, improvement coexisted with residual uncertainty.

Participant Quotes: "We don't know what will happen next."

## Discussion:

The findings reflect the multidimensional burden mothers experience when caring for their child with leukaemia. Emotional distress at diagnosis and the gradual journey toward the adjustment mirror existing as global evidence [10]. Mothers' intensive involvement in infection control, nutrition and treatment adherence aligns with earlier studies showing that they disproportionately shoulder the caregiving demands for the sake of their children [11]. Financial strain, the disrupted routines and social withdrawal are consistent with literature describing the challenges faced by families in low- resource settings, where inadequate support systems aggravate the caregiver burden [12]. Their reliance on the personal coping mechanisms highlights the caregiver resilience, but also underscores the limited availability of structured psychosocial services [13, 14]. Supportive communication from the healthcare professionals, particularly the nurses, played a central role in the enhancing mothers' confidence consistent with studies on caregiver-provider relationships [15]. Persistent apprehension about the relapse underscores the long-term psychological vulnerability reported in certain paediatric cancer caregiving research [16].

## Conclusion:

Mothers caring for their children with leukaemia experience a profound emotional, social and practical challenge that shape their caregiving role. Despite immense strain, they demonstrate remarkable resilience, sustained by the emotional bonding, vigilant care practices and selective support from their family and the healthcare providers. Integrating the structured nurse-led psychosocial interventions emphasising the need for counselling, stress management, education and continuity of communication could significantly enhance the caregiver well-being and caregiving capacity.

## Implications for practice:

[1] Establish nurse-led counselling programmes focusing on the emotional expression, coping strategies and the education on disease trajectory.

[2] Develop structured training modules on the infection control, nutrition, home-based symptom management and safe play activities.

[3] Introduce caregiver support groups to reduce isolation and stigma.

[4] Strengthen continuity of care through dedicated oncology nurse coordinators.

[5] Provide financial guidance and resource linkage to reduce economic strain.

## Figures and Tables

**Table 1:** Themes, subthemes and participant transcripts

**Theme**	**SubthemeParticipant Transcripts**
Inner Emotional Storm	1.1 Emotional Turmoil	"I was scared when I saw my baby." - P1
		"I couldn't see her." - P1
		"We were crying every day." - P4
	1.2 Emerging Positivity	"We accepted it little by little." - P6
		"Doctor said improvement is there." - P2
		"We believe he will recover." - P6
	1.3 Persistent Worry	"That's what I am worried about." - P1
		"I am afraid her leave will end." - P1
		"He is afraid he might hurt himself." - P5
Pathway to Diagnosis	2.1 Tests & Referral	"Blood level was low." - P2
		"WBC is too high." - P3
		"After bone marrow, they confirmed." - P5
	2.2 Realisation & Acceptance	"We didn't know this disease existed." - P2
		"Only after they explained, we understood." - P6
		"After that we accepted." - P6
	2.3 Diagnostic Uncertainty	"We thought it was dengue or typhoid." - P6
		"Fever stopped and again started." - P6
		"Further tests were needed." - P2
Caregiving Patterns	3.1 Hygiene & Infection Prevention	"I use sanitizer." - P1
		"We sanitize before touching him." - P1
		"We don't allow many people." - P3
	3.2 Diet & Nutrition	"I don't buy outside food." - P1
		"Food only from home." - P3
		"I prepare porridge and rice." - P5
	3.3 Play & Routine Care	"Play keeps him active." - P2
		"They taught me how to play." - P5
		"I keep him clean daily." - P5
Psychosocial and Practical Consequences	4.1 Financial Burden	"I am the one paying the bill." - P1
		"I stopped tailoring work." - P4
		"He misses work to stay here." - P3
	4.2 Family Role Redistribution	"My mother helps me." - P1
		"My husband supports me." - P6
		"My daughter takes care of me." - P1
	4.3 Disrupted Daily Life	"We suffered a lot sir." - P5
		"I don't know what to do." - P1
		"Everything is on me." - P5
Social Support and Challenges	5.1 Social Withdrawal	"We don't attend functions." - P2
		"We stopped taking him out." - P5
		"We keep distance." - P1
	5.2 Selective Disclosure	"We say only blood is low." - P3
		"We keep it in family." - P4
		"We don't tell anyone." - P3
	5.3 Community Support	"My husband comes daily." - P1
		"My relatives helped me." - P4
		"My mother stayed with me." - P1
Stress-Care Strategies	6.1 Emotional Bonding	"I spend time with my kids." - P1
		"They will be relaxed." - P1
		"I feel calm." - P1
	6.2 Mindfulness & Calmness	"Mindful meditation… I understood." - P6
		"Relaxation was useful." - P6
		"I'm doing per your advice." - P1
	6.3 Routine Tasks as Coping	"I like cooking." - P1
		"I cook at home." - P1
		"Cooking makes me feel better." - P4
Healthcare Relationship Dynamics	7.1 Nurse Support	"Everything Sir said was useful." - P6
		"Nurse cleared doubts." - P6
		"They taught cleanliness." - P5
	7.2 Doctor Communication	"Doctor said improvement is there." - P2
		"Doctor told me baby won't survive." - P1
		"Doctor explained after blood test." - P3
	7.3 Hospital Logistics	"We came by ambulance." - P1
		"I was in ICU." - P1
		"Doctor referred to Egmore." - P6
Perceived Therapeutic Outcomes	8.1 Following Instructions	"We follow everything." - P2
		"We follow what you told." - P1
		"We wash hands." - P6
	8.2 Visible Improvement	"He is 50% cured." - P2
		"Is it better to go home?" - P1
		"Improvement is there." - P2
	8.3 Continued Worries	"We don't know what's happening." - P3
		"I'm worried." - P1
		"Her leave will end." - P1
